# A Case of Metropolypus

**Published:** 1864-07

**Authors:** D. B. Trimble


### A CASE OF METROPOLYPUS.

By D. B. TRIMBLE, M. D.

*Editors of Chicago Medical Journal—Gentlemen:*

Though there is nothing extraordinary in the following case, or probably in its treatment, yet a record of the successful management of any severe case of disease, where the remedies resorted to are evidently beneficial, may be instructive to the younger, perhaps encouraging to the more experienced, practitioner; and I therefore, venture to send for publication, if you think it worthy of a place in your journal, the following case of Metropolypus:

Miss ——, a lady about 40 years of age, of very delicate health, and who had previously been under my care for several years, for various affections, called to consult me for a tumor in the vagina which caused her much uneasiness. She had been affected with uterine hæmorrhage, at intervals, for some months; retention, of urine, often requiring the use of the catheter; and pain in the loins. A few years previous to this period she lost a near relative with cancer of the womb, and was impressed with the belief that she was similarly affected, as she complained of a feeling of weight and fulness in the pelvis, and was confident of the presence of a tumor. Her nervous system was in an exceedingly excited condition, and had been so for some years—being subject to such severe neuralgic pains as to throw her into violent spasms. One of the seats of pain was at the symphisis pubis, the frequency and severity of which was latterly much increased.

On examination I found a tumor about three inches in length by one and a half inches in diameter, of a firm consistence, and insensible to the touch. The tumor would sometimes recede more than half its length, and, when the patient was recumbent, would nearly disappear. I had no doubt of it being a polypus, of the fibrous variety, and so informed her, and also of the necessity of an operation. This she was willing to endure, and requested me to perform it, but never having had a similar case, I preferred having a more experienced operator, and sent for an eminent surgeon from Philadelphia. In the meantime, to prevent it from receding, I tied a ligature of very narrow tape as high up on the tumor as possible, and secured it to her garter.

The operation was performed two days after (while under the influence of ether), with Gooch's canula, and the wire applied external to the os tincæ. In about 80 hours I removed the canula with the tumor. The odor was intolerable for several days, and there continued to be a semi-sanguineous discharge for about two weeks. The patient continued to feel pain in the loins and pelvic region for several weeks, which gradually subsided. From the size and shape of the tumor (its diameter being nearly alike throughout its length), from the quantity and quality of the discharge, and from the continued suffering, I felt convinced that the tumor had not all been extirpated, and expected that, in time, the operation, which was performed in July, 1862, would have to be repeated.

In September following, she informed me that she felt confident that the tumor was re-forming, but she was able to keep about until the following April, when she sent for me, and upon making a vaginal examination I found a tumor nearly filling the vagina, more fleshy than the last, and somewhat pyriform. No pedicle could be felt, however, but at the os uteri, which firmly compressed it; its diameter was about an inch Being a lady of intelligence I explained to her, and the friend who nursed his, that the tumor had not descended sufficiently to make an operation effect a radical cure; that it was necessary, to accomplish this, to ligate the neck of the polypus, to perform which, the os uteri must be dilated to allow of the further transmission of the tumor; that the womb itself must, if possible, be made to expel it, and that, for this purpose, I would be obliged for a time, probably for some days, to place her under the influence of suitable medicines.

Viewing the uterus as in a condition analogous to that in the puerperal state, and that its fibres might be made to contract, I administered ergot in moderate and repeated doses, which, in less than twenty-four hours, produced considerable pain. As the os tincæ was still rigid I covered it with the ext. belladona, and at my next visit found the os was somewhat relaxed and the tumor lower. Continued the treatment, but on the third day, owing to the earnest entreaties of the patient, I consented to operate, though the stem of the polypus was still within the os. This, however, must have been near, from its diminished diameter. I had procured a long Gooch's canula, and Museux's forceps, and after bringing her fully under the influence of ether, I passed the canula nearly an inch within the os uteri, (it being dilated and dilatable,) and applied the ligature with very little difficulty. On the fifth day I made (as I had done for the two previous days) some traction on the canula with a view to removing it, but it adhered too firmly to enable me to do so. I then applied the forceps and used as much force as I thought proper, but without success. About an hour after I left, however, she got out of bed when the whole mass, with the instrument, came away, its weight breaking the slight attachment which had prevented me from removing it. I had no means at hand to weigh it, but it measured seven and a half inches in circumference and four in length. There was but little discharge subsequently, and much less fetor than in the previous operation, and which disappeared in a few days and did not return. Though I was not satisfied that I had grasped the neck of the tumor by the wire, yet I felt that it was in all probability a radical cure; up to April 6th, 1864, there has been no appreciable return of the disease.

The points of interest in this case are, that in the first operation, it was performed too soon and without sufficient preparation, so that a portion of the tumor was left; and in the second, that the contractile efforts of the womb can be excited even in its unimpregnated state, sufficient for useful practical purposes. How far the belladonna was useful in dilating the os it is difficult to determine, for slight contractile efforts, but no dilation, had commenced before its application. I think it had an influence, and at any rate can do no injury. The severe pains that this patient suffered can not, in any great degree, be attributed to the polypus, for, as before observed, she had been a sufferer from various causes for a number of years. But the acute neuralgic pain in the symphisis pubis was, if not caused, much aggravated by it, for since the last operation, she has suffered comparatively little in this region.

When the disease has advanced to the degree that affected my patient, there is of course no means of relief except chirurgical, but the question arises, can the causes of the disease be counteracted and its production prevented?

From the secluded condition of the parts affected; from the absence of pain, at least until the tumor attained such a size as to prevent the possibility of a medical cure; and from our want of knowledge of the condition of the system predisposing to the disease, it is very difficult to discover it in time to administer the proper remedies—did we know them. The question presents, must the system be in a sthenic or asthenic condition to produce polypii? It is evident that our therapeutics will depend upon our decision. In my patient, though she was much emaciated, there was no approach to anæmia, or to an asthenic condition; and from a case reported by Dr. Ramsbotham; and others that have been reported, I arrive at the conclusion that the uterus is in a hypertrophic or sthenic state when polypi are produced. An asthenic system could not eliminate such immense morbid growths as we read of or witness. In Dr. Ramsbotham's case the patient, who was in labor, had a polypus projecting from the uterus, "as large as a goose's egg," and which was forced from the vagina by the parturient throes of the womb forcing the child's head against it. The woman having been delivered, it became a question, what should be done with the polypus? In consultation with his father, they declined removing it for the following, among other reasons, "Again, it seemed likely that, as the adventitious growth was nourished by the same vessels that supplied the uterus, these vessels had become enlarged in proportion somewhat equivalent to the increase in the calibre of the uterine vessels themselves. If such were the case, it was fair to infer that, as the uterine vessels shrunk after delivery, the vascularity of the polypus would also be materially diminished; and that this diminution in the bulk of the morbid growth would render its removal altogether less formidable."

We can have no doubt that the uterus is in an exalted or sthenic condition, when in the gravid state, and the ratiocination of the Drs. Ramsbotham was certainly correct. The result of the case fully justified this opinion, for he says, "After the lapse of nearly four months, no symptoms appearing in the meantime to call for earlier inteference, the polypus was tied in the usual manner, and sloughed off in five days; and at the time of its removal its size was scarcely so great as a walnut divested of its outer husk." This rapid reduction in the size of the tumor shows that as the active condition of the womb abated, the exciting cause of the polypus was lessened; affording another proof that the sthenic state of the organ was essential to the rapid development of the tumor.

The greatest difficulty in these cases is that of diagnosing them in the earlier stages, as there is then so little derangement of the general system, or at least it is so indefinitely known, that the symptoms are uncertain. But if there are sufficient reasons to suspect the existence of a case, or if it is in parts where it can be early ascertained, as in the vagina, urethra, or nares, remedial treatment may avail. In this event probably a moderate anti-phlogistic course, low diet, antimonials, etc., in connection with the iodides, bromides, etc., might be successful. Dr. Stewart, of Peekskill, N. Y., records a case of large "ovarian tumor," which was treated with bromide of potassium with very great alleviation, and the general effect of these remedies would seem to indicate their usefulness in this disease. Where there is great prostration or anæmia produced by hemorrhage, the reducing plan would, of course, be contra-indicated, and tonics, stimulants, and nourishing diet should be administered to give tone to the system, and local applications made to check hemorrhage.

But I am much inclined to the opinion of Professor Meigs, that medicines are of but little avail in the treatment of polypi. "If this view of the case be just," he says, "it would be idle to attempt to modify or control the growth of such a tumor, by means of drugs or medicines administered internally, since, however powerfully such drugs or medicines might be able to modify the actions of the woman's constitution under its natural physiological laws, they could not reach in their influence, nor in any degree control the accretion forces employed in the production of polypus, which, being heterologue, will not, neither, indeed, can come under obedience to the physiological law of the woman's life; a law with which it has no longer any lot or part, beyond that of living as long as she lives, preying as a parasite upon her materials, and sending back no answering organic influences to serve in maintaining that beautiful harmony of the organisms whose concert of action is life. Such a growth is not a part or parcel of the economy, it is not like one of the members of a family or a flock, but like a stranger, or a thief and a robber, that has entered in by guile or violence, to dwell among them, and to disturb and destroy them. Hence you see that such tumors are to be treated chirurgically and not medically. They may be extirpated, they can not be cured. Save yourselves and your patients, therefore, the trouble and loss of using physic or drugs."

